# Inhibiting Heat Shock Protein 90 (HSP90) Limits the Formation of Liver Cysts Induced by Conditional Deletion of *Pkd1* in Mice

**DOI:** 10.1371/journal.pone.0114403

**Published:** 2014-12-04

**Authors:** Zachary B. Smithline, Anna S. Nikonova, Harvey H. Hensley, Kathy Q. Cai, Brian L. Egleston, David A. Proia, Tamina Seeger-Nukpezah, Erica A. Golemis

**Affiliations:** 1 Yale University, New Haven, Connecticut, 06520, United States of America; 2 Program in Molecular Therapeutics, Fox Chase Cancer Center, Philadelphia, Pennsylvania, 19111, United States of America; 3 Synta Pharmaceuticals, Lexington, Massachusetts, 02421, United States of America; 4 Department I of Internal Medicine, Center for Integrated Oncology, University Hospital of Cologne, Cologne, 50937, Germany; University of Cincinnati, College of Medicine, United States of America

## Abstract

Polycystic liver disease (PLD) occurs in 75–90% of patients affected by autosomal dominant polycystic kidney disease (ADPKD), which affects 1∶400–1,000 adults and arises from inherited mutations in the *PKD1* or *PKD2* genes. PLD can lead to bile duct obstructions, infected or bleeding cysts, and hepatomegaly, which can diminish quality of life. At present, no effective, approved therapy exists for ADPKD or PLD. We recently showed that inhibition of the molecular chaperone heat shock protein 90 (HSP90) with a small molecule inhibitor, STA-2842, induced the degradation of multiple HSP90-dependent client proteins that contribute to ADPKD pathogenesis and slowed the progression of renal cystogenesis in mice with conditional deletion of *Pkd1*. Here, we analyzed the effects of STA-2842 on liver size and cystic burden in *Pkd^-/-^* mice with established PLD. Using magnetic resonance imaging over time, we demonstrate that ten weeks of STA-2842 treatment significantly reduced both liver mass and cystic index suggesting selective elimination of cystic tissue. Pre-treatment cystic epithelia contain abundant HSP90; the degree of reduction in cysts was accompanied by inhibition of proliferation-associated signaling proteins EGFR and others, and induced cleavage of caspase 8 and PARP1, and correlated with degree of HSP90 inhibition and with inactivation of ERK1/2. Our results suggest that HSP90 inhibition is worth further evaluation as a therapeutic approach for patients with PLD.

## Introduction

Autosomal dominant polycystic kidney disease (ADPKD), an inherited syndrome affecting 1∶400–1,000 individuals [1], [2], arises from mutations in the *PKD1* or *PKD2* genes, encoding the polycystins. ADPKD is invariably associated with the replacement of normal kidney parenchyma with fluid-filled cysts in middle-aged adults. For many individuals with APDKD, a secondary feature of the disease is the development of hepatic cysts [3], [4], [5], which can be symptomatic or asymptomatic. Polycystic liver disease (PLD) has been associated with mutations in both the *PKD1* and *PKD2* genes in patients, and is also observed in genetically engineered mice bearing these mutations [6], [7], [8]. Those who suffer from PLD and ADPKD typically develop renal failure and require dialysis and/or kidney transplantation, but rarely require hepatic transplantation. However, some individuals can experience PLD-associated complications including infected and bleeding cysts, bile duct obstruction and hepatomegaly that can require surgical intervention and diminish quality of life.

The polycystin proteins encoded by *PKD1* and *PKD2* regulate multiple signaling pathways that influence hepatic and renal growth and homeostasis. In ADPKD, renal cells have multiple anomalously activated signaling proteins relevant to these processes, including ribosomal protein S6 (S6), ribosomal S6 kinase (RSK/S6K), AKT, mammalian target of rapamycin (mTOR), SRC, ERK1/2, and RAF, among others [1], [2]. Therapeutics that have been evaluated for the treatment of ADPKD include targeted inhibitors of some of these proteins, such as SRC and mTOR [9], [10]. These have shown some potential for improvement of symptoms in preclinical models [11]. In clinical trials, mTOR inhibitors have demonstrated some effect in slowing kidney growth, although have had less pronounced effect on kidney function [12]. However, no highly effective therapy is currently available [13]. While many features of growth control in hepatic and renal cells are conserved and similarly affected by mutations associated with ADPKD, there is some evidence the biology of cyst formation differs in the two organs (reviewed in [14]). Somatostatin analogs have offered some benefit in reducing liver cystogenesis [15], as has inhibition of mTOR or VEGFR [7], [8].

In attempting to improve management of ADPKD, we considered that numerous studies of drug effectiveness in cancer have indicated that inhibiting a single signaling protein is generally insufficient for halting tumor growth because of functional redundancy in pathways [16], [17]. Many of the signaling proteins activated in ADPKD are also commonly activated in cancer [18], and notably, many of these proteins are dependent on the molecular chaperone heat shock protein 90 (HSP90) for stability and/or activity. HSP90 inhibition has recently demonstrated particular clinical efficacy in cancer, based on the simultaneous inhibition of multiple pro-proliferative proteins in the absence of this important chaperone [19]. In recent work, we found that inhibition of HSP90 significantly slowed renal cystogenesis and kidney growth *in vivo* in mice developing ADPKD because of a conditional knockout of the *Pkd1* gene [20]. Because of this encouraging result, we hypothesized that HSP90 inhibition might also be beneficial for controlling the growth of hepatic cysts. In this study, we assessed the efficacy of the HSP90 inhibitor STA-2842 in limiting the development of PLD in conditional *Pkd1*-knockout mice. Our results, presented below, suggest that HSP90 inhibition may be both a useful and a well-tolerated therapeutic approach for patients with PLD.

## Results and Discussion

**Figure 1:**
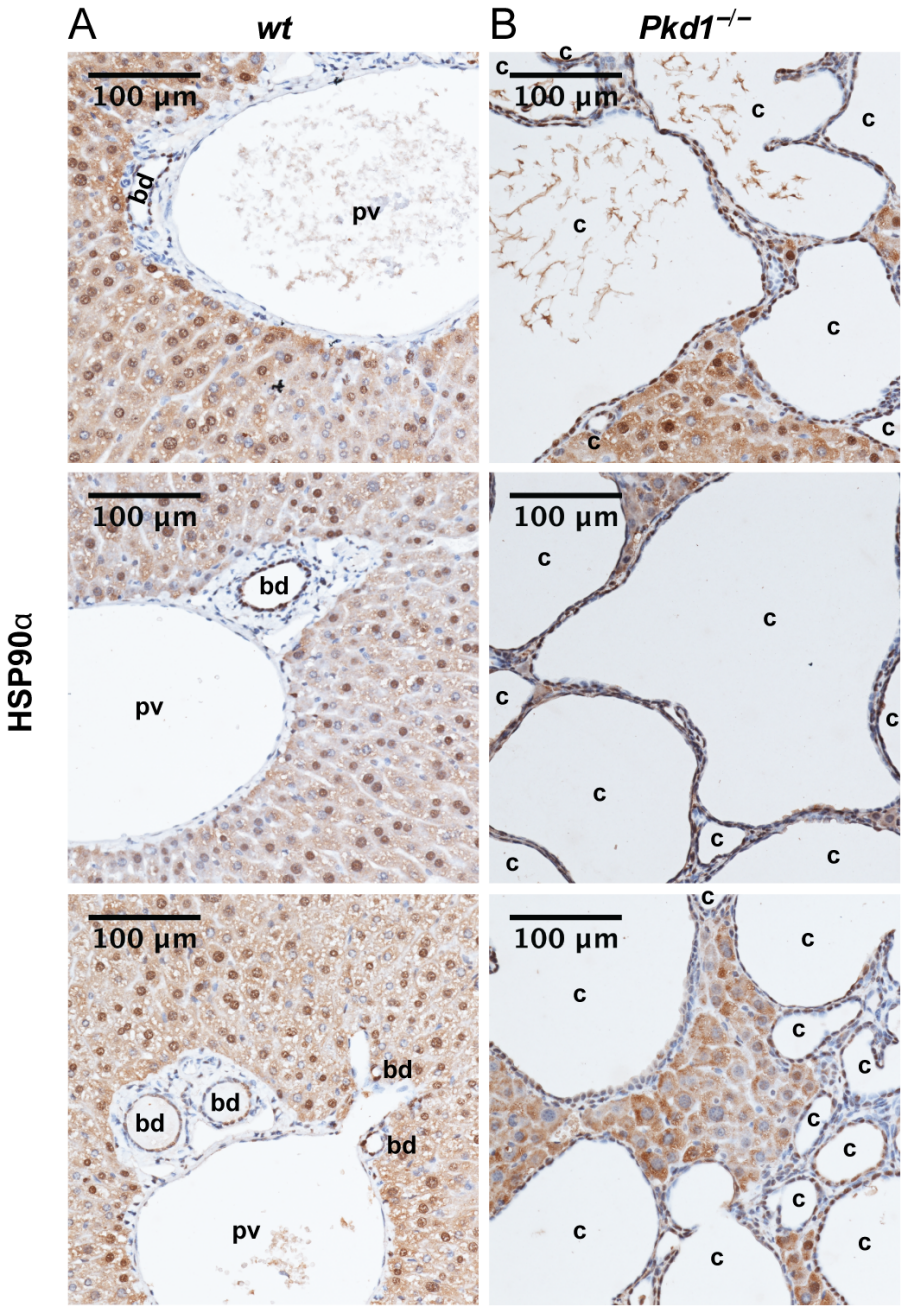
HSP90α is upregulated in epithelial cells lining liver cysts. (**A, B**) Representative hematoxylin stained liver sections with immunohistochemical detection of HSP90α (brown) from three (**A**) wild type (wt) and (**B**) *Pkd1* ^–/–^ mice. bd  =  bile duct, pv  =  portal vein, c  =  cyst. Scale bars  = 100 µm; magnification, 20x.

To assess the value of HSP90-targeting inhibitors for PLD, we first analyzed HSP90 expression in cystic livers. Conditional *Pkd1* knockout mice (*Pkd1fl/fl;Cre/Esr1^+^*, subsequently labeled *Pkd1^−/−^*) and control mice (*Pkd1fl/fl;Cre/Esr1^−^*, subsequently labeled wild type, *wt*) were injected with tamoxifen at post-natal days P37 and P38 of age to inactivate *Pkd1*, leading to significant hepatic cystogenesis [21], [22]. Immunohistochemical analysis of liver sections from 6.5-month-old mice with established PLD indicated consistent and intense expression of the inducible isoform HSP90α in the epithelial cells lining liver cysts in *Pkd1^−/−^* mice (**Fig. 1**). No endothelial cells lining blood vessels (portal veins) in either *Pkd1^−/−^* or wt mice expressed HSP90α. Bile ducts observed in either genotype displayed heterogenous expression of HSP90α, with some having moderate to high staining, but others negative. In non-cystic tissue, low levels of HSP90α staining were observed in hepatocytes (**Fig. 1**).

**Figure 2:**
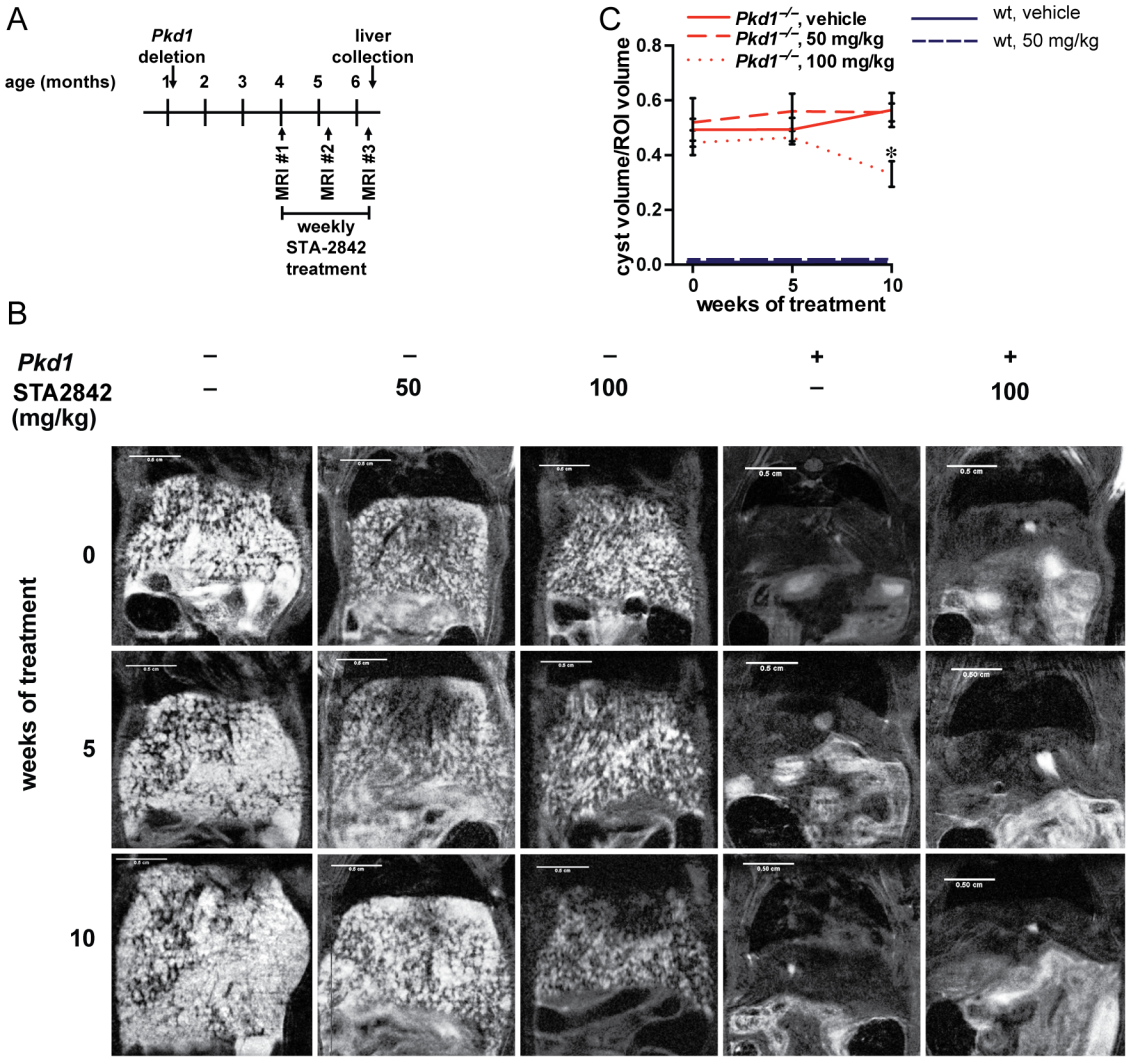
HSP90 inhibition reduces late stages of liver cyst formation in conditional *Pkd1* ^–/–^ mice. (**A**) Schematic outlining the timetable of the study. (**B**) Representative MRI images of mice, imaged at 0, 5, and 10 weeks of treatment with vehicle or drug (STA-2842) in *wt* (+) or *Pkd1* ^–/–^ (–) mice. Scale bars  = 0.5 cm. (**C**) Cyst volume estimated as a percentage of hepatic tissue at 0, 5, and 10 weeks of treatment of *Pkd1* ^–/–^ or wt mice. *n* = 6–8 mice. * indicates comparisons to *Pkd1* ^–/–^, vehicle-treated mice: *, *P*≤0.05. All data were graphed as mean ± standard error of the mean (SEM). No cysts were detected in any wt mice.

We analyzed whether the HSP90 inhibitor STA-2842, a resorcinolic triazole molecule that selectively binds with the N-terminus on the ATP binding pocket of HSP90 [20], controlled the growth of liver cysts. After tamoxifen treatment at P37/P38, *Pkd1^−/−^* and *wt* mice were dosed weekly for 10 weeks with STA-2842 (50 mg/kg and 100 mg/kg) or vehicle (5% dextrose) starting at 4 months of age (**Fig.2A**). Magnetic resonance imaging (MRI) was performed at 0, 5, and 10 weeks after the commencement of treatment to longitudinally track the development of hepatic cysts (**Fig. 2B**). Hepatic cysts were already abundant in 4-month-old mice, and did not further increase in volume over the following 10 weeks in mice treated with vehicle (**Fig. 2C**). MRI analysis indicated no change in liver cyst volume in *Pkd1^−/−^* mice treated with 100 mg/kg STA-2842 for 5 weeks, however; a significant decrease in cyst volume was observed after 10 weeks of treatment (∼41%; p = 0.01). Low dose STA-2842 (50 mg/kg) had no statistically significant effect on liver cystogenesis at either time point (**Fig.2C**).

**Figure 3:**
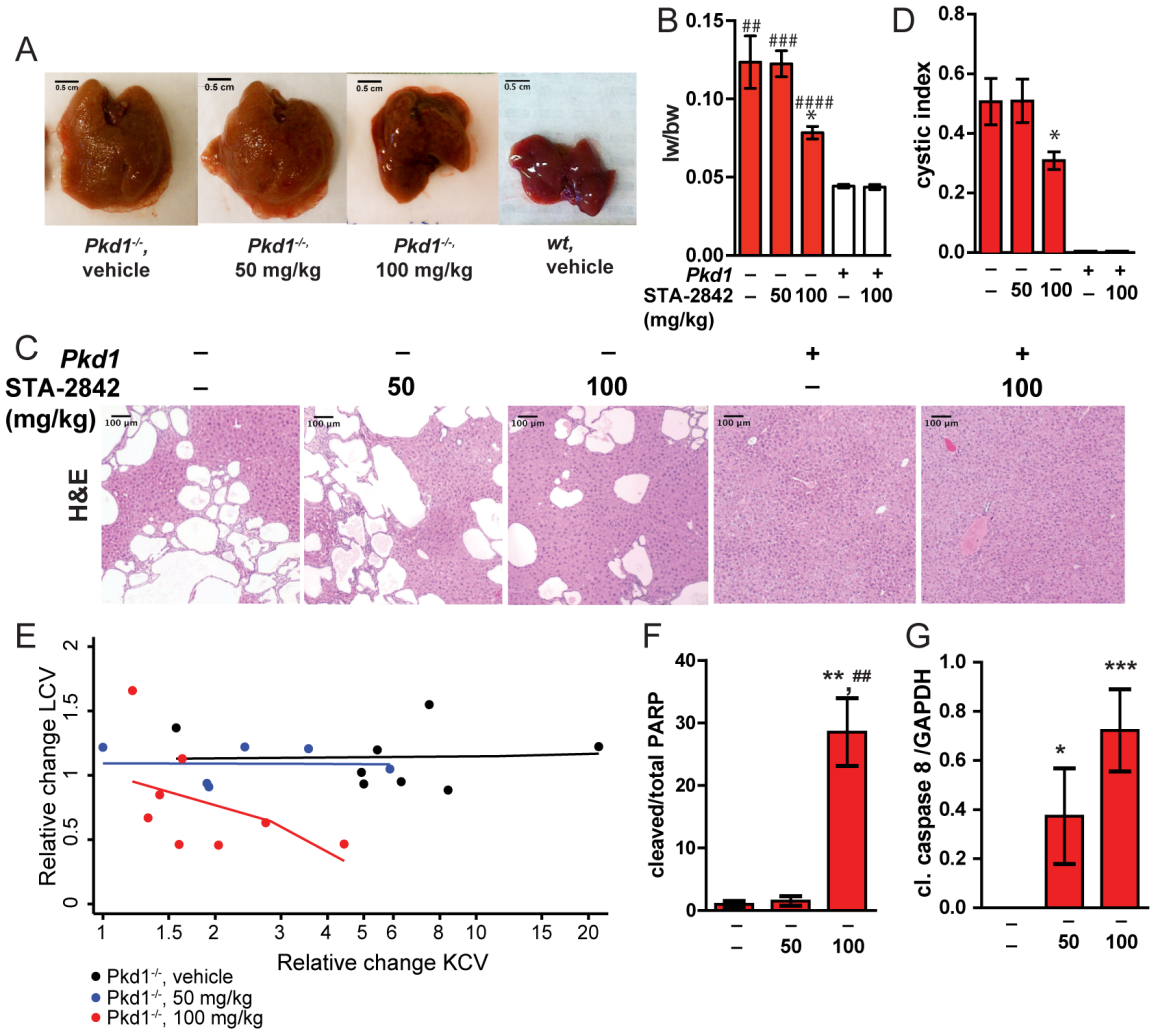
Histopathological development of cysts. (**A**) Representative livers collected from *Pkd1* ^–/–^ or *wt* mice treated with vehicle or STA-2842 at the indicated doses, at 6.5 months of age. (**B**) Liver weights (lw) to body weight (bw) ratio of in *wt* (+) or *Pkd1* ^–/–^ (–) mice after 10 weeks of treatment with STA-2842 (50, 100) or vehicle (–). *n* = 5–8 mice. * indicates comparisons to *Pkd1* ^–/–^, vehicle-treated mice; # indicates comparisons to *wt* vehicle-treated mice: *, *P*<0.05; ^##^, *P*<0.01; ^###^, *P*<0.001; ^####^, *P*<0.0001. (**C**) Representative H&E stained liver sections after 10 weeks of treatment with vehicle or drug in *wt* (+) or *Pkd1* ^–/–^ (–) mice, indicating extent of cystogenesis. Scale bars  = 100 µm; magnification  = 10x. (**D**) Cystic indices quantified as a percentage of grid intersections that cross cysts on H&E slides. *n* = 6–8 mice. * indicates comparisons to *Pkd1^–/–^*, vehicle-treated mice: *, *P*<0.05. All data graphed as mean ± standard error of the mean (SEM). (**E**) Relative change in cyst burden by drug dose (6 month value divided by 4 month value), with simple linear fits of the relationship shown. None of the slopes were statistically significant (p>0.11 in all three cases). Since the range of values differed among the three doses, the X-axis is drawn on the log scale to better depict the relationships. **F**. Relative expression of cleaved PARP, normalized to total PARP, following treatment of *Pkd1* ^–/–^ (–) mice with the indicated doses of STA-2842. **, *P*<0.01 to vehicle-treated mice; ^##^, P<0.01 relative to mice treated with 50 mg/kg STA-2842. **G**. Relative expression of cleaved caspase 8, normalized to GAPDH, following treatment of *Pkd1* ^–/–^ (–) mice with the indicated doses of STA-2842. *, *P*<0.05, ***, P<0.001 in reference to vehicle-treated mice.

After 10 weeks of treatment, mice were euthanized and livers analyzed (**Fig. 3A, B**). Liver weight of vehicle treated mice was significantly enlarged in vehicle treated *Pkd1^−/−^* mice in contrast to wt mice (179% increase; *P* = 0.002). However, the size of *Pkd1^−/−^* livers was very significantly decreased (36.5%; *P* = 0.0316) in mice that had been treated with 100 mg/kg of STA-2842, while no effect was seen with 50 mg/kg STA-2842 (**Fig. 3A, B**), while crude visual inspection indicated a qualitative difference in color and texture of the livers treated with 100 mg/kg STA-2842, versus the other two cohorts. Hematoxylin and eosin (H&E) staining of liver sections confirmed the reduced cyst burden predicted by MRI imaging (**Figs. 3C, D**). Quantification predicted a ∼39% decrease in the percentage of grid intersections with cystic tissue in H&E stained slides, as compared to vehicle treated *Pkd1^−/−^* mice, but no noticeable decrease with the 50 mg/kg dose.

Activity of STA-2842 in reducing liver cysts could be independent of activity in the drug in controlling expansion of kidney cysts; alternatively, reduction of cysts in the two organs may be correlated. To evaluate these possibilities, we compared cyst burden in liver and kidney tissue from [20], across the three treatment groups (**Fig. 3E**, **Fig. S1**). This analysis indicated that first, there is no correlation between the burden of cysts in the kidney and the liver cross-sectionally before dosing commences (**Fig S1**). Second, there is no correlation for drug response between 4 and 6 months at the 0 or 50 mg/kg doses. While at the 100 mg/kg dose a slight negative relationship is observed, it is not statistically significant (p = 0.12) (**Fig. 3E**). These results suggest that if there is a relationship of kidney cysts with liver cysts, the association is weak at best.

A reduction in cystic and liver volume likely reflects changes in proliferation or apoptosis induced by STA-2842. In the analysis of liver sections collected at the experiment endpoint and stained for the proliferation marker Ki-67 and the apoptotic marker cleaved caspase 3 (**Fig. S2**), less than 1% of nuclei were positive for either marker making it impossible to quantify an effect, and consistent with the fact that cystic diseases such as PLD are progressive and slow-acting. However, examination of tissue lysates by Western blot (**Fig. S3**) with antibodies to total and cleaved PARP (**Fig. 3F**) and cleaved caspase 8 (**Fig. 3G**) indicated STA-2842 increased basal levels of apoptosis, in a dose-dependent effect.

**Figure 4:**
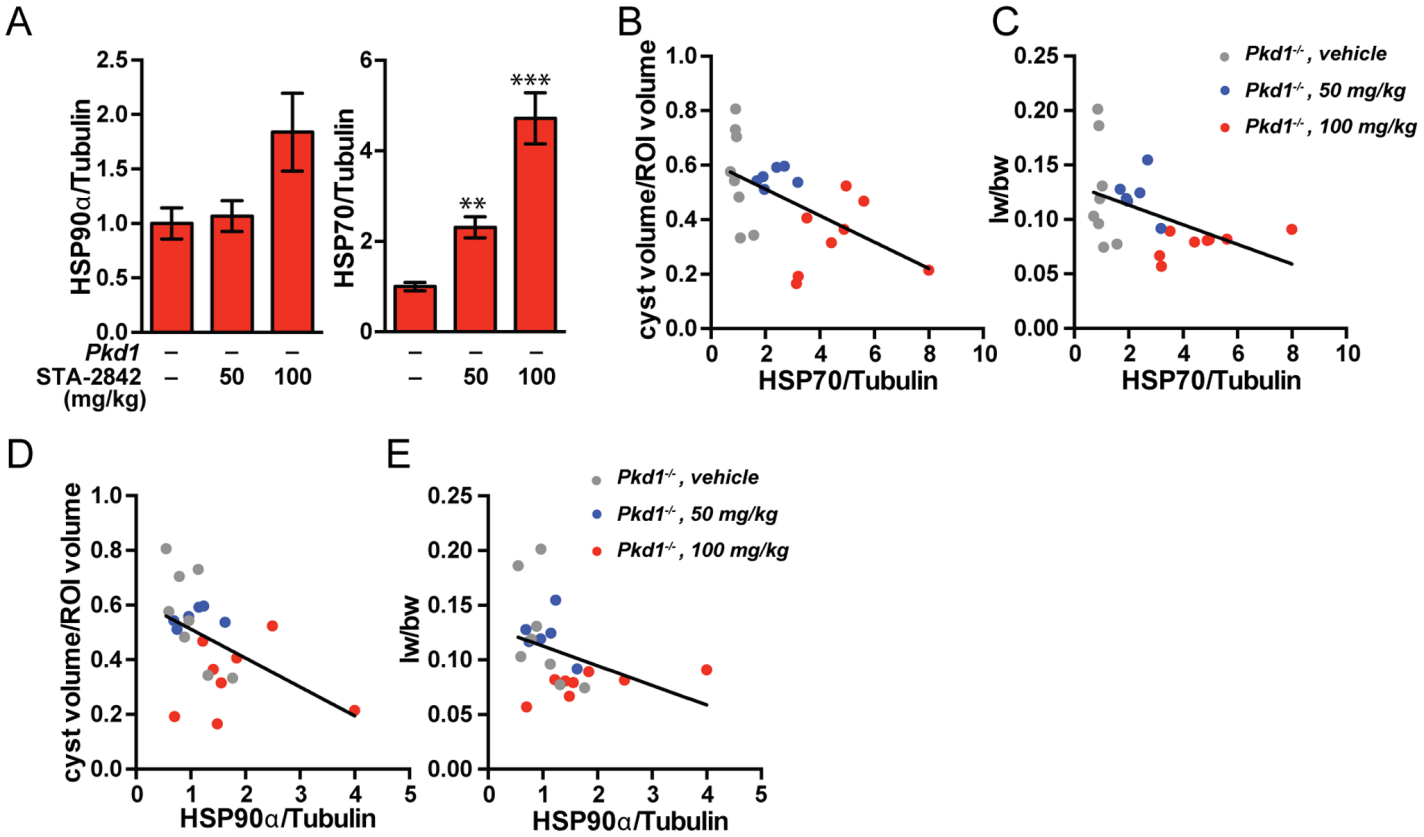
Changes in protein expression and activation of heat shock proteins associated with HSP90 inhibition. (**A**) Quantification of HSP90α and HSP70 expression levels among the *Pkd1* ^–/–^ groups. *n* = 6–8 mice. * indicates comparisons to *Pkd1* ^–/–^, vehicle-treated mice: **, *P*≤0.01; ***, *P*≤0.001. (**B–E**) Negative correlations between HSP70, HSP90α expression in the drug treatment groups and (**B, D**) cyst volume/region of interest (ROI) (*P* = 0.0046 for HSP70 and *P* = 0.0136 for HSP90) and (**C, E**) liver weight/body weight (lw/bw) ratios (*P* = 0.0141 for HSP70 *P* = 0.0138 for HSP90). Dots represent individual mice; lines represent linear regression functions. *n* = 6–8 mice.

To gain insight into the role of STA-2842 in influencing proliferative signaling pathways in the context of cystogenesis, we analyzed liver lysates by Western analysis for the expression and/or activity of HSP90 and other relevant proteins (**Fig S3**). We first looked for evidence of HSP90 inhibition, which is typically reflected by compensatory upregulation of an alternative heat shock protein, HSP70 [23], and occasionally by elevated expression of total HSP90. We observed a trend toward upregulation of HSP90 at the 100 mg/kg dose level of STA-2842, and statistically significant, dose-dependent upregulation of HSP70 (**Fig. 4A**). We then compared expression of HSP70 (**Figs. 4B, C**) and HSP90 (**Figs. 4D, E**) with the cyst volume and liver weight to body weight ratio for all individual mice in the treatment groups. This indicated a striking negative correlation between the expression of either heat shock protein with both cystic volume and liver weight, indicating that degree of efficacy of the STA-2842 compound in individual mice predicted degree of therapeutic response, and compatible with the observation that response in kidney and liver cysts was correlated at the effective 100 mg/kg dose (**Fig. 3E**).

**Figure 5:**
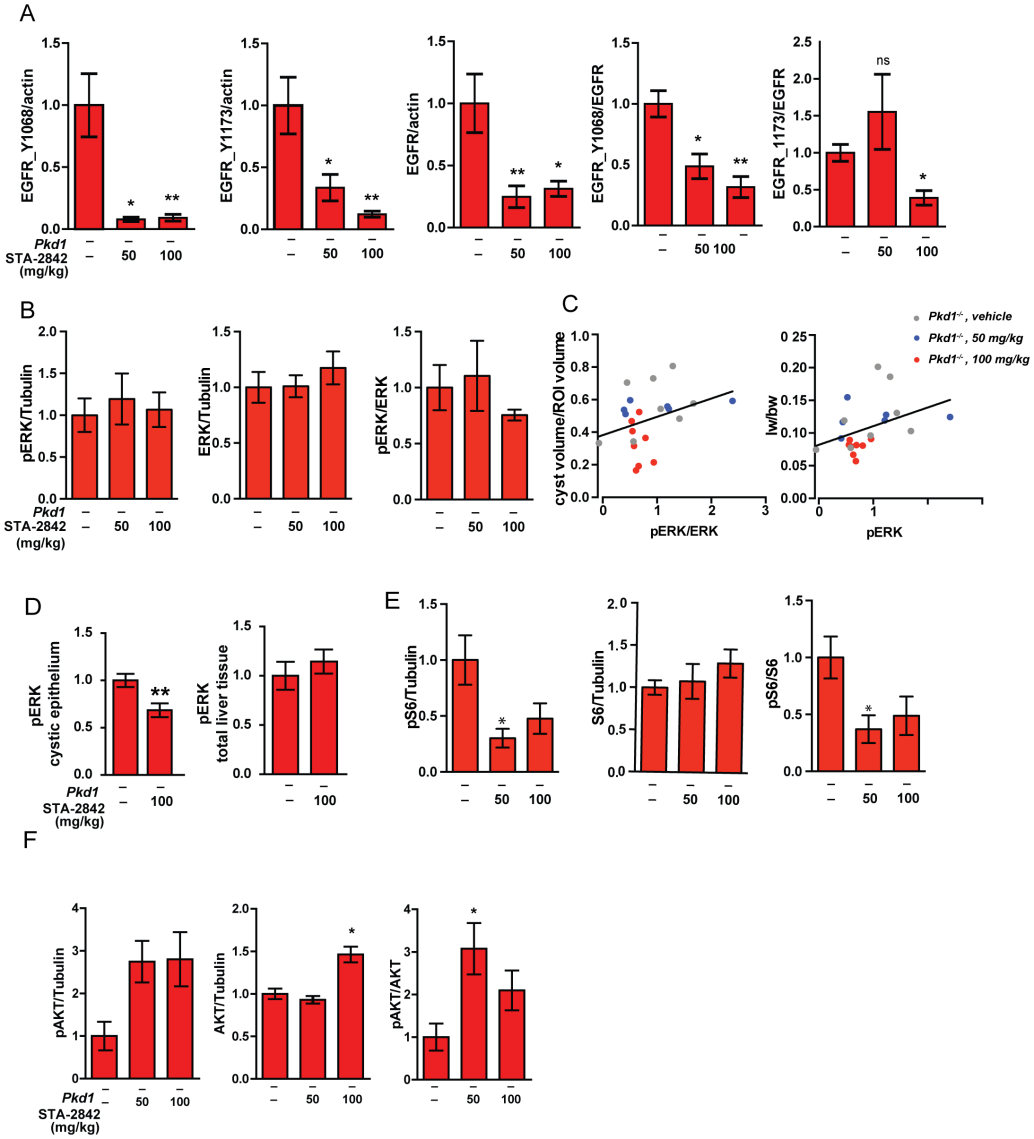
Changes in protein expression and activation of signaling proteins associated with HSP90 inhibition. (**A**) Differences in expression of phosphorylated Y^1068^ and Y^1173^ EGFR and total EGFR normalized to actin, and Y^1068^ and Y^1173^ EGFR normalized to total EGFR, following treatment with the indicated doses of STA-2842. *, P<0.05; **, P<0.01. (**B**) Insignificant differences in expression of phosphorylated T^202^/Y^204^ ERK1/2 or total ERK1/2, normalized to tubulin, and phosphorylated T^202^/Y^204^ ERK1/2 normalized to total ERK1/2, following treatment with the indicated doses of STA-2842; differences are not significant (**C**) Positive correlations between the ratio of phosphorylated T^202^/Y^204^ ERK1/2 normalized to total ERK1/2 expression in the drug treatment groups and (left) cyst volume/region of interest (ROI) (*P* = 0.174) and (right) liver weight/body weight (lw/bw) ratio (*P* = 0.040). Dots represent individual mice; lines represent linear regression functions. *n* = 6–8 mice. All data graphed as mean ± standard error of the mean (SEM). (**D**) Expression of phosphorylated T^202^/Y^204^ ERK1/2 in cystic epithelia (left) or total liver (right) quantified from immunohistochemical staining of liver sections from *Pkd1* ^–/–^ mice treated with vehicle or 100 mg/kg STA-2842 (see Fig S4). **, P<0.01. (**E**) Differences in expression of phosphorylated phS^235^/S^236^ S6 or total S6, normalized to tubulin, and phosphorylated phS^235^/S^236^ S6 normalized to total S6, following treatment with the indicated doses of STA-2842; *, P<0.05. *P-*values for comparisons between *Pkd1* ^–/–^ vehicle- versus 100 mg/kg-treated groups bordering on significant (*P* = 0.060). (**F**) Differences in expression of phosphorylated phS^473^ AKT or total AKT, normalized to tubulin, and phosphorylated phS^473^ AKT normalized to total AKT, following treatment with the indicated doses of STA-2842; *, P<0.05.

We next analyzed by Western analysis the expression and activation of two proteins strongly linked to the increased proliferation of PLD cells, EGFR, ERK1/2, the ribosomal protein S6, and AKT (**Fig. 5** **; Figs. S3** **and** **S4**). As EGFR, Akt1, the ERK1/2 activator RAF, and the S6 activator mTOR are all direct clients of HSP90, STA-2842 would be expected to reduce expression and/or activity of these proteins [20]. STA-2842 reduced expression of total EGFR, and additionally reduced the ratio of phY^1068^/total and phY^1173^/total EGFR at the effective 100 mg/kg dose (**Fig. 5A**). Treatment with STA-2842 reduced the ratio of active (T^202^/Y^204^-phosphorylated) to total ERK1/2 at the 100 mg/kg dose level, although this was not statistically significant (**Fig. 5B**). However, in correlative analysis, mice with the lowest ration of phosphorylated to total ERK had a significantly reduced liver weight to body weight ratio (**Fig. 5C**), implying biological importance of the reduction. Subsequent analysis of phT^202^/Y^204^ERK1/2 in cystic epithelial linings versus total liver tissue indicated a significant reduction in the epithelial cells, although not in total liver tissue (**Fig 5D**, **Fig. S4**). Based on all of these analyses, inhibition of the EGFR-ERK1/2 signaling axis at the 100 mg/kg dose corresponded well to biological inhibition of cyst growth. Finally, in further Western analysis, STA-2842 treatment significantly reduced activation (phS^235^/S^236^ phosphorylation) of the S6 protein, while not affecting total expression of S6, at both the 50 mg/kg and 100 mg/kg dose levels (**Fig. 5E**). Levels of active phS^473^ AKT and total AKT were somewhat elevated, as was the phS^473^/total AKT ratio, following STA-2842 treatment, but this response was also not dose-dependent and significant (**Fig. 5F**).

In sum, these results indicate that HSP90 facilitates PLD pathogenesis and provide a proof of concept for the exploration of HSP90 inhibition to treat PLD. Importantly, this represents the first observation that treatment with an HSP90 inhibitor can reverse established disease. This was not observed in the response of renal cysts to STA-2842, where the main effect of the drug was to limit the rate at which new cysts were formed [20]. The likely reason for the difference in outcome is the greater regenerative capacity of hepatic versus renal tissue. Although the overall low levels of proliferation of hepatic cystic tissue make it difficult to quantify drug action, the significant inhibitory effect of HSP90 inhibition on ERK and EGFR activity at the effective dose, with ERK inhibition particularly concentrated in cystic epithelia, generally support this interpretation.

STA-2842 is a preclinical agent, which is not likely to move into the clinic in the near future. However, a related compound, ganetespib (formerly STA-9090), has demonstrated encouraging signs of clinical activity in breast cancer and ALK+ lung cancer [19], and is currently undergoing late stage clinical trials, with little evidence of toxic side effects. While particular care would be required to ensure HSP90 inhibition by ganetespib does not cause cytotoxicity specifically in the setting of PLD, where liver function is compromised, the data presented here suggest that it may be worth evaluating ganetespib in this disease. The range of therapeutic possibilities for HSP90 inhibitors extends beyond their sole use as a single inhibitor to remedy disease pathology. Some research suggests that HSP90 inhibition can help sensitize the body to chemotherapies, rendering it useful in treating cancers driven by multiple or indeterminate initiating factors [24], [25]. In a randomized study of docetaxel with or without ganetespib in advanced stage lung adenocarcinoma patients, the combination of ganetespib and docetaxel showed an acceptable safety profile and demonstrated substantial improvements in both progression free survival and overall survival [26]. Additionally, using HSP90 inhibitors in combination with other targeted protein inhibitors may be beneficial. In ADPKD and PLD pathogenesis, increased expression and activation of mTOR-related signaling proteins is important [7]. HSP90 inhibition has already been shown to combine productively with mTOR inhibitors in cancer settings [23]. This result suggests that pairing an HSP90 inhibitor with an mTOR inhibitor such as everolimus or temsirolimus may bring about an increased therapeutic effect, revivifying interest in an mTOR inhibitory strategy that was less promising than expected in initial clinical trials [27].

## Materials and Methods

### Ethics statement

All mouse work was approved by the Institutional Animal Care and Use Committee (IACUC) of Fox Chase Cancer Center (animal protocol #06-1). The Animal Welfare Assurance on file in the Office of Laboratory Animal Welfare is #A3285-01. All mice were handled by individuals with appropriate specialized training. Mice were anesthetized during MRI scanning and were euthanized by CO_2_ asphyxiation.

### Mouse strains and drug treatment

Conditional *Pkd1* knockout mice were created utilizing the Cre-flox regulatory system for specific inactivation of the *Pkd1* gene *in vivo* and were kindly supplied by Gregory Germino (NIDDK, National Institutes of Health, Bethesda, MD) [21], [22]. To inactivate *Pkd1*, *Pkd1fl/fl;Cre/Esr1^+^* (*Pkd1^−/−^*) mice and *Pkd1fl/fl;Cre/Esr1^−^* (wild type, *wt*) mice were intraperitoneally injected with a tamoxifen (250 mg/kg, resolved in corn oil) on postnatal days P37 and P38 to induce *Pkd1* deletion in the non-control mice. Both male and female specimens were used in all mouse groups, and gender was determined not to affect the results of the experiments. Mice were dosed weekly for 10 weeks starting at 4 months of age with vehicle (5% dextrose), or 50 mg/kg or 100 mg/kg of STA-2842. Mice were euthanized at ∼6 months of age by CO_2_ asphyxiation. Livers were collected, weighed, and divided, with part placed in 10% phosphate-buffered formaldehyde, and part flash frozen.

### MRI administration and image analysis

Mice were imaged at 0, 5, and 10 weeks of treatment in a vertical bore MR system, using methods described in detail previously [20]. The ImageJ (NIH, Bethesda, MD) program was used to quantify cyst volume measurements. A method of subtracting backgrounds then thresholding MRI images was used to approximate the area of the white (i.e. cystic) areas in the livers within an oval region of interest (ROI) of 0.178 cm^2^ on a single MRI. The ROI was selected so that cysts in hepatic tissue could be analyzed over 7 sequential images. To establish the % cystic tissue, the location of cysts between MRIs was assumed to be constant for the purposes of this analysis and the total cystic area was multiplied by the distance between the images (0.075 cm) and then divided by the total volume the cylindrical ROI (0.09345 cm^3^).

### Protein expression analysis by fluorescent Western blotting

Flash frozen tissue from *Pkd1^-/-^ mice* was lysed and protein concentrations of the resulting cystic liver lysates were measured using the Pierce BCA Protein Assay Kit (Thermo Scientific, Waltham, MA). Lysates were denatured at 98°C in sample buffer for 10 minutes, and 42 µg of protein was loaded in NuPAGE Novex 8% Bis-Tris 20 Well Midi Protein Gels (Life Technologies). Two SDS-PAGE gels containing all analyzed specimens were run in parallel in an XCell SureLock Mini-Cell Electrophoresis System (Life Technologies, Grand Island, NY), with 4 samples common to both gels as a reference group. The proteins were then transferred overnight at 20 V from to Immobilon-FL PVDF membranes (EMD Millipore, Billerica, MA). The membranes were incubated for 40 minutes at room temperature in a blocking solution of a 1∶1 mix of phosphate buffered saline (PBS) containing 0.1% Tween 20 (Sigma-Aldrich, St Louis, MO), to Odyssey blocking Buffer (LI-COR). Odyssey Blocking Buffer (LI-COR, Lincoln, NE). The membranes were then incubated for 1 hour at room temperature or overnight at 4°C in primary antibodies (1∶1000): anti-HSP70 (monoclonal, mouse, #ADI-SPA-820) or anti-HSP90α (polyclonal, rabbit, #ADI-SPS-771) (both from Enzo, Farmingdale, NY); or anti-pERK1/2^Thr202/Tyr204^ (polyclonal, rabbit, #9101), anti-ERK1/2 (monoclonal, mouse, #4696), anti-pS6^Ser235/236^ (monoclonal, rabbit, #4858), anti-S6 (monoclonal, mouse, #2317), anti-phospho AKT^S473^ (polyclonal, rabbit, #4060S) and anti-AKT (polyclonal, rabbit, #2920S) and anti-α/β-Tubulin (polyclonal, rabbit, #2148) (each from Cell Signaling, Danvers, MA). IRDye800CW Goat anti-Mouse and IRDye 680 RD Goat anti-Rabbit secondary antibodies (1∶10,000, LI-COR, city, state) were applied at room temperature for 1 hour. Membranes were imaged and quantified in separate channels (700 and 800 nm) in the Odyssey Infrared Imaging System using Odyssey V3.0 software (LI-COR).

To analyze the expression levels of phospho- and total EGFR, PARP and cleaved caspase 8, cells were lysed and resolved by SDS-Page. Western Blotting was performed using standard procedures, and blots developed by chemiluminescence using Luminata Western HRP substrates (Classico, Crescendo and Forte, EMD Millipore) and Immun-Star AP Substrate (Bio-Rad Laboratories). Primary antibodies included anti-phospho EGFR^Y1068^ (polyclonal, rabbit, #3777S, Cell Signaling Technologies), anti-phospho EGFR^Y1173^ (polyclonal, rabbit, #44794G, Invitrogen) and anti-EGFR (polyclonal, rabbit, #2646, Cell Signaling Technologies), anti-PARP (polyclonal, rabbit, #9542S, Cell Signaling Technologies), anti-cleaved caspase 8 (polyclonal, rabbit, #8592P, Cell Signaling Technologies), anti-GAPDH (mouse, monoclonal mAbcam 9484, #ab9482, Abcam) and anti-vinculin (mouse, monoclonal hVIN-1, #V9131, Sigma-Aldrich). Secondary anti-mouse and anti-rabbit HRP-conjugated antibodies (GE Healthcare) were used at a dilution of 1∶10,000 and secondary anti-mouse and anti-rabbit AP-conjugated antibodies (Jackson Immunoresearch Labs) were used at a dilution of 1∶5,000. Quantification of signals on Western blots was done using the NIH ImageJ Imaging and Processing Analysis Software with signaling intensity normalized to loading control.

### Tissue preparation, histology, and immunohistochemistry

Sections of the mouse livers were suspended in formalin (10% phosphate-buffered formaldehyde) for 1–2 days and were subsequently dehydrated and embedded in paraffin blocks. Paraffin blocks were prepared into slides stained by hematoxylin and eosin (H&E), anti-Ki-67 (1∶100, monoclonal rat anti-mouse, #M7249, Dako, Carpinteria, CA), anti-HSP90α (1∶800, monoclonal rabbit, #NB120-2928, Novus Biologicals, Littleton, CO), anti-pERK1/2^Thr202/Tyr204^ (polyclonal, rabbit, #9101, Cell Signaling, Danvers, MA), or cleaved caspase-3 (1∶200, polyclonal, #9661 Cell Signaling, Danvers, MA) using standard procedures described previously [20]. All slides containing immunohistochemical staining were imaged using a ScanScope CS scanner (Aperio, Leica Biosystems, Buffalo Grove, IL). To quantify cystic indices, the ImageJ program was used to generate computerized grids with an area per point of 5000 µm^2^ and to superimpose them upon images of H&E-stained liver sections scanned at 10x magnification using an EVOS XL Core Cell Imaging System (Life Technologies, Grand Island, NY). Cystic indices were calculated as a percentage of grid intersections that crossed cysts, with ∼1450 grid intersections analyzed for per mouse. Clear areas with diameters >40 µm were defined as cysts; those with diameters >20 µm defined as dilated tubules.

### Statistical analysis

We used *t*-tests, assuming unequal variances for hypothesis testing. Prism 6 (GraphPad Software, San Diego, CA) was used to identify statistical correlations between protein expression and physical results and then to plot regression lines. We used nonparametric Spearman correlations to examine relationships between variables of interest, such as relative change in kidney and liver cyst volumes between time points.

## Supporting Information

Figure S1 **Cyst burden in liver versus kidney tissue at the month 4 baseline (p = 0.57).**
(TIF)

Figure S2 **Absence of Ki-67 and cleaved caspase-3 among treated and non-treated** ***Pkd1*** **^–/–^mice.** Representative hematoxylin stained liver sections with immuno-histochemical detection of (**A**) Ki-67 (brown) and (**B**) cleaved caspase-3 (brown) from three independent *Pkd1* ^–/–^(–) mice treated with vehicle, 50 mg/kg STA-2842, or 100 mg/kg STA-2842. Magnification, 20x. Scale bars  = 200 µm.
(TIF)

Figure S3 **Western blotting for proteins indicated, from** ***wt*** **(+) or** ***Pkd1*** **^–/–^ (–) mice after 10 weeks of treatment with STA-2842 (50, 100 mg/kg) or vehicle (–).**
(TIF)

Figure S4 **Representative hematoxylin stained liver sections with immunohistochemical detection of phosphorylated T^202^/Y^204^ ERK1/2 (brown) from** ***Pkd1*** **^–/–^ mice treated with vehicle or 100 mg/kg STA-2842, as indicated.** Scale bar – 50 µm.
(TIF)
