# Supplementary figures and images for: Inhibiting Heat Shock Protein 90 (HSP90) Limits the Formation of Liver Cysts Induced by Conditional Deletion of Pkd1 in Mice
Source: PLoS One. 2014 Dec 4;9(12):e114403. doi: 10.1371/journal.pone.0114403 (PMC4256400; doi:10.1371/journal.pone.0114403)

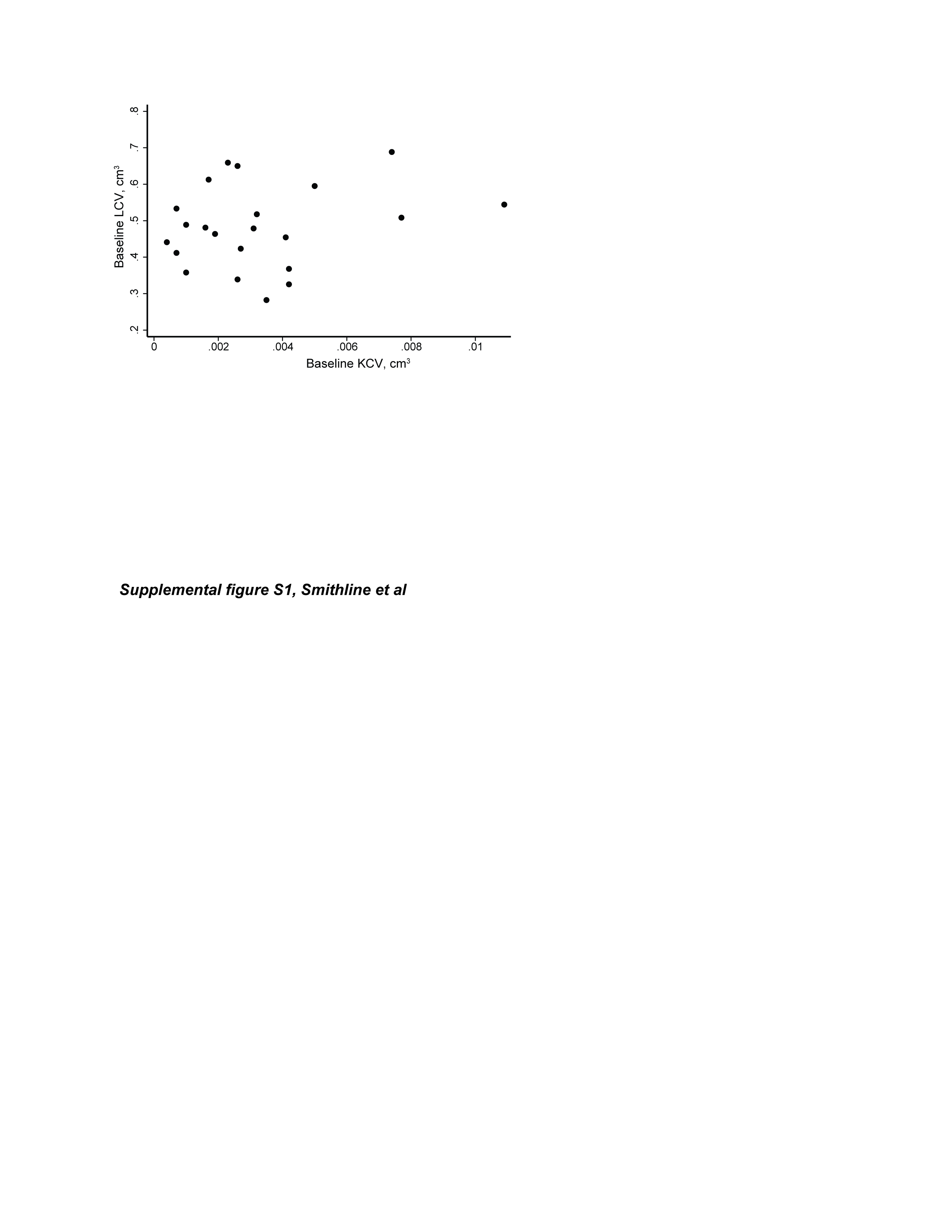

Supplement: Figure S1 — Cyst burden in liver versus kidney tissue at the month 4 baseline (p = 0.57). (TIF) [file pone.0114403.s001.tif]

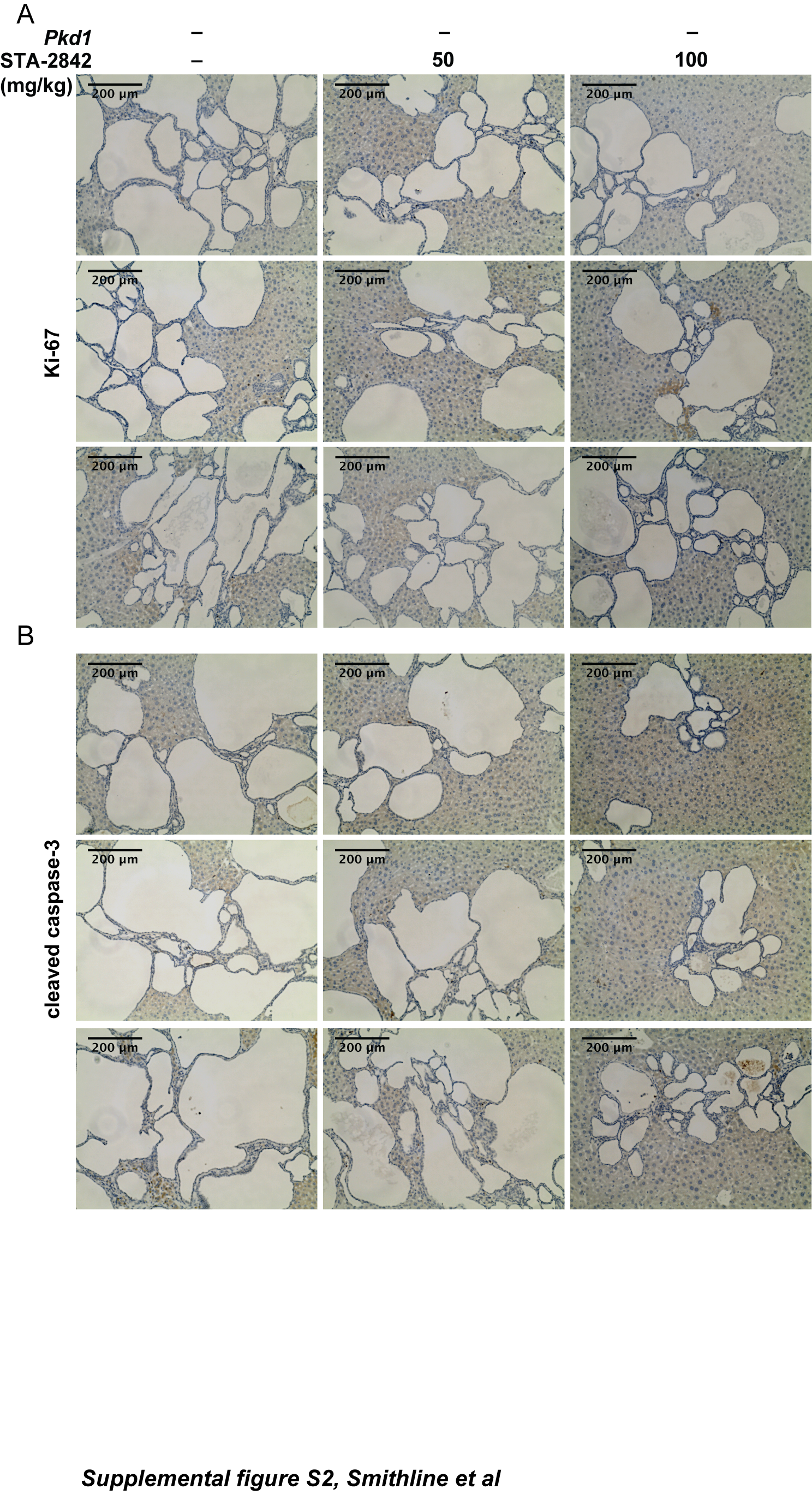

Supplement: Figure S2 — Absence of Ki-67 and cleaved caspase-3 among treated and non-treated Pkd1 –/–mice. Representative hematoxylin stained liver sections with immuno-histochemical detection of (A) Ki-67 (brown) and (B) cleaved caspase-3 (brown) from three independent Pkd1 –/–(–) mice treated with vehicle, 50 mg/kg STA-2842, or 100 mg/kg STA-2842. Magnification, 20x. Scale bars = 200 µm. (TIF) [file pone.0114403.s002.tif]

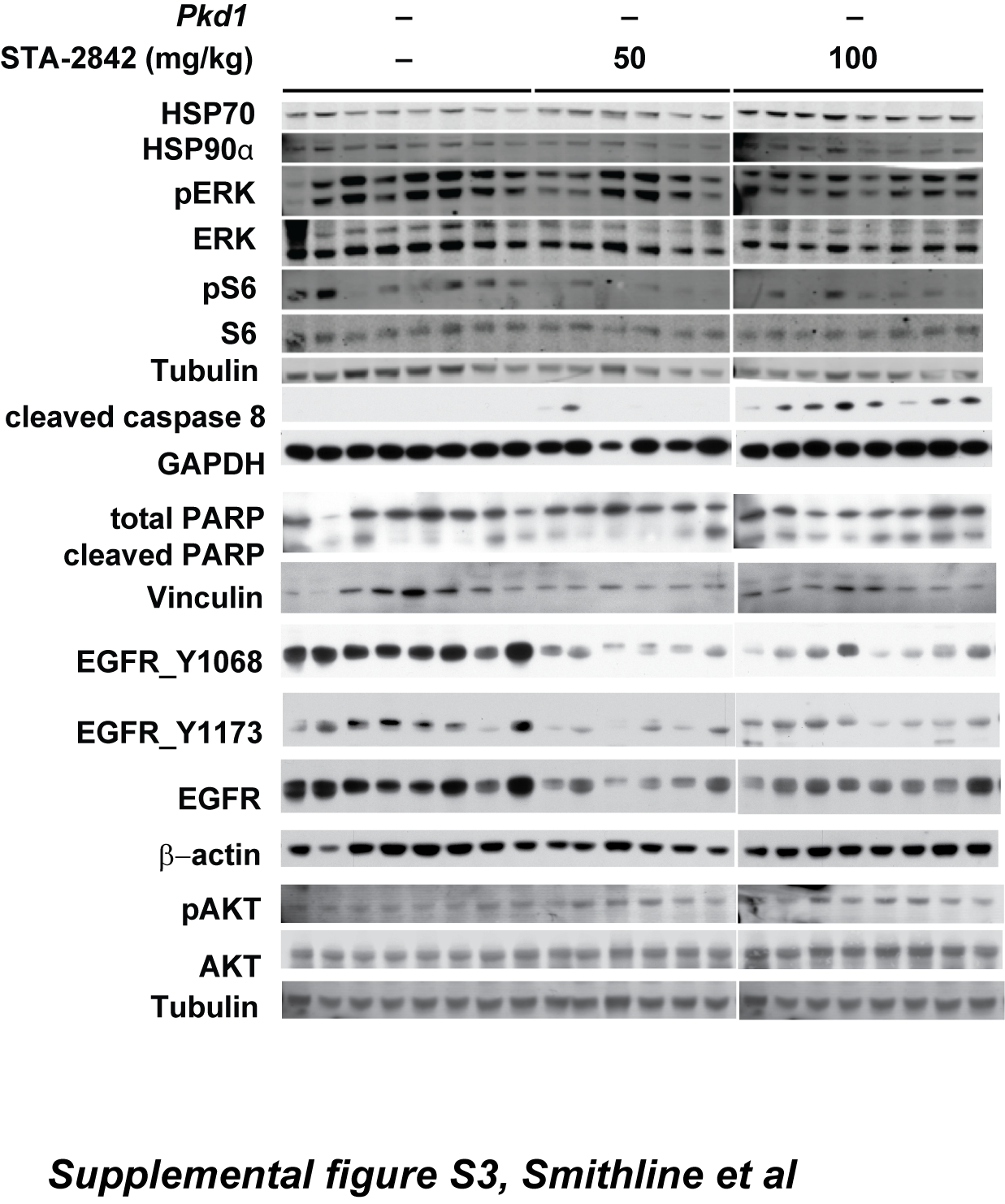

Supplement: Figure S3 — Western blotting for proteins indicated, from wt (+) or Pkd1 –/– (–) mice after 10 weeks of treatment with STA-2842 (50, 100 mg/kg) or vehicle (–). (TIF) [file pone.0114403.s003.tif]

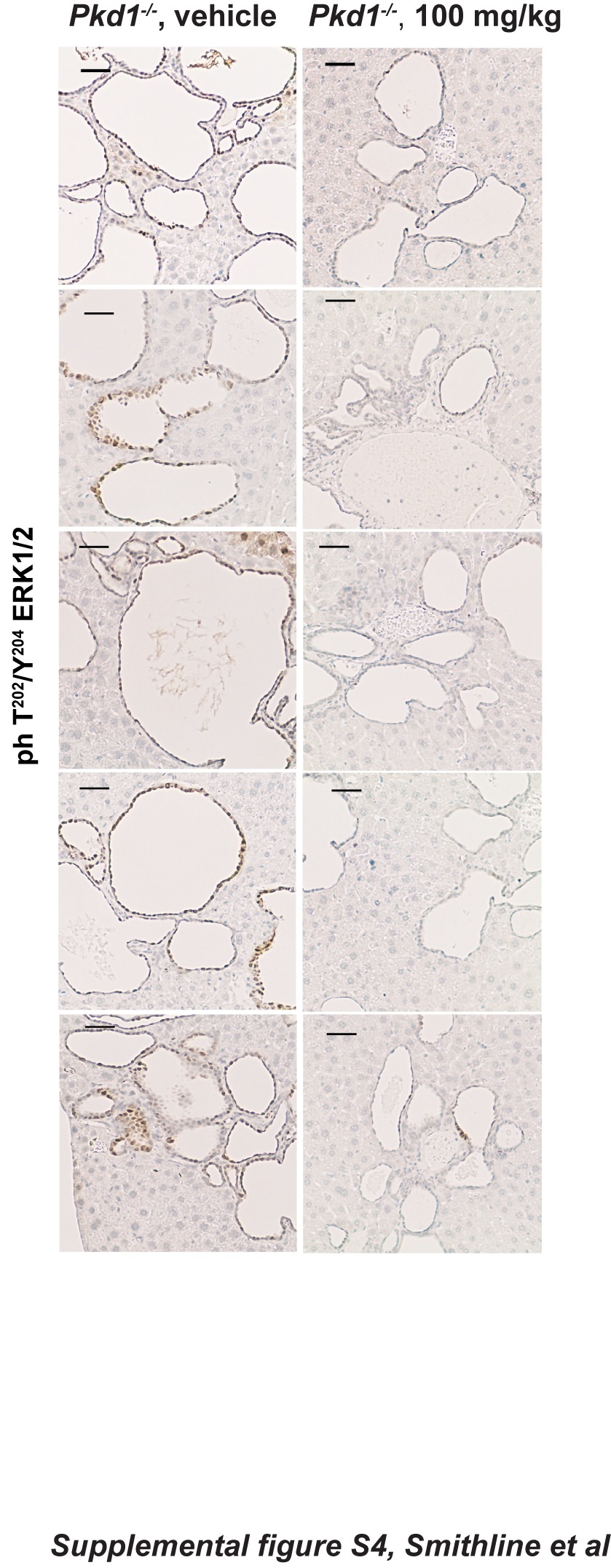

Supplement: Figure S4 — Representative hematoxylin stained liver sections with immunohistochemical detection of phosphorylated T202/Y204 ERK1/2 (brown) from Pkd1 –/– mice treated with vehicle or 100 mg/kg STA-2842, as indicated. Scale bar – 50 µm. (TIF) [file pone.0114403.s004.tif]
